# Single-Molecule Phosphorescence and Intersystem Crossing in a Coupled Exciton Plasmon System

**DOI:** 10.1021/acsnano.5c04193

**Published:** 2025-06-25

**Authors:** Abhishek Grewal, Hiroshi Imada, Kuniyuki Miwa, Miyabi Imai-Imada, Kensuke Kimura, Rafael Jaculbia, Klaus Kuhnke, Klaus Kern, Yousoo Kim

**Affiliations:** † Max-Planck-Institut für Festkörperforschung, 28326 Heisenbergstrasse 1, Stuttgart 70569, Germany; ‡ 13593 Surface and Interface Science Laboratory, RIKEN, Wako, Saitama 351-0198, Japan; § 13592 Institute for Molecular Science, Myodaiji, Okazaki, Aichi 444-8585, Japan; ∥ Institut de Physique, École Polytechnique Fédérale de Lausanne, Lausanne 1015, Switzerland

**Keywords:** phosphorescence, scanning tunneling microscopy, intersystem crossing, plasmonics, STM-induced luminescence, tip-enhanced fluorescence

## Abstract

Scanning the sharp metal tip of a scanning tunneling microscope (STM) over a molecule allows for tuning the coupling between the tip plasmon and a molecular fluorescence emitter. This allows access to local variations in fluorescence field enhancement and wavelength shifts, which are central parameters for characterizing the plasmon-exciton coupling. Performing the same for phosphorescence with molecular-scale resolution remains a significant challenge. In this study, we present the investigation of phosphorescence from isolated Pt-Phthalocyanine molecules by analyzing tip-enhanced emission spectra in both current-induced and laser-induced phosphorescence. The latter directly monitors singlet-to-triplet state intersystem crossing of a molecule below the tip. The study contributes to a detailed understanding of triplet excitation pathways and their potential control at submolecular length scales. Additionally, the coupling of organic phosphors to plasmonic structures is a promising route for improving light-emitting diodes.

## Graphical abstract

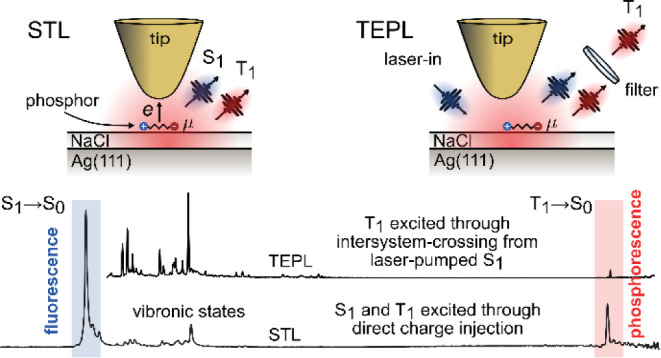

## Introduction

Elucidating the processes occurring within isolated luminescent molecules has witnessed significant progress in recent years. Detailed studies have been carried out using STM metal-tip-enhanced molecular electrofluorescence^1–10^ and photofluorescence^11–17^ with atomic scale precision. Extending this experimental approach to phosphorescence with plasmonic tip enhancement has turned out to be a significant challenge because of low intensity and debated assignments of observed emission lines. Phosphorescence, the emission resulting from an excited triplet state transitioning to a ground singlet state, is a nominally forbidden transition and thus cannot be directly excited by light. However, this transition can become observable via “intensity borrowing” when triplet and singlet states mix, for example, through spin–orbit coupling (SOC). Excitation of triplet states occurs either by intersystem-crossing (ISC) from a higher-lying singlet state (which can be directly excited by light absorption) or by charge injection. ISC is a process operating on the molecular states in the opposite direction as up-conversion electroluminescence (UCEL), which has garnered increasing interest in single-molecule STM studies recently.^18–21^ Moreover, molecular excitation through electron injection with random spin as employed in organic light emitting diodes (OLEDs) can generate spin-triplet (T_1_) and spin-singlet (S_1_) states in a 3:1 ratio.^22^ Harnessing the otherwise lost energy of the triplet state occupancy is a major motivation for incorporating organic phosphors into host layers of electroluminescent OLEDs, kick-starting the search for suitable triplet emitters.^23^ Recently, decay rate enhancement via coupling to plasmonic systems^24–26^ in OLEDs has shown improved device stability^27^ against aging.^28,29^ For this reason, studying the influence of plasmon coupling on relative fluorescence and phosphorescence intensities, as well as ISC, at the single-molecule level is of great interest.

In this context, it is surprising that the direct observation of phosphorescence in close proximity to a metallic tip remained elusive for a long time. Early studies addressing this issue remain under debate, as the emission line was attributed either to phosphorescence^30^ or to fluorescence from a charged molecular state.^8,16,31^ A subsequent investigation^32^ revealed that both states lie extremely close in energy, preventing definitive assignment. Questions also persist regarding the relative intensities of “forbidden” phosphorescence with respect to dipole-allowed transitions occurring between energetically higher-lying states. Due to the longer lifetime of the triplet state, its branching ratio to radiative decay is assumed negligible for a molecule in an STM and thus in proximity to a conductor. However, there is consensus that the lowest triplet state plays a very crucial role as a relay state in UCEL^20,21,33^ and is essential for a comprehensive understanding of intramolecular excitation dynamics.^34^

To avoid ambiguities, we present a study of a molecule known for its strong phosphorescence emission. We use an STM (operated at a temperature of 4.3 K in vacuum at pressures <10^–11^ mbar) equipped with an optical detection system that enables tip-enhanced optical spectroscopy with atomic resolution. A low coverage of molecules of platinum­(II) phthalocyanine (PtPc) is evaporated in ultrahigh vacuum (UHV) on top of few monolayers (ML) of NaCl epitaxially grown on an Ag(111) surface. The NaCl layer reduces the luminescence quenching that may occur due to hybridization with substrate electronic states.^35^ PtPc is a well-suited candidate for this study due to its intense phosphorescence,^36–39^ attributed to strong SOC^37^ facilitated by its heavy central Pt atom. The molecule provides an excellent platform for studying exciton–plasmon (X–P) interaction^2,9,40^ in conjunction with ISC and phosphorescence, leading to a favorable condition for the observation of phosphorescence.^37,41–43^ In this study, we report strong fluorescence from PtPc and also investigate its phosphorescence, which remained previously unobserved in low-temperature STM experiments.^33^ We further investigate how the properties of fluorescence and the coupling to plasmons modify the observed phosphorescence intensity. Finally, we provide clear evidence of ISC at the single-molecule level using tip-enhanced photoluminescence,^11,12^ while avoiding the direct excitation of phosphorescence through charge injection into the molecule.

## Results and Discussion

### STM-Induced Electroluminescence (STML) of a Single Phosphor

For details of sample preparation and experimental procedures see the Supporting Information 1.1. PtPc molecules adsorb in planar geometry on NaCl, with their four isoindole units aligned along the [100] and [010] directions of the NaCl lattice (Figure 1A), and with the metal center positioned at a Na site (inset of Figure 1A). Differential conductance (d*I*/d*V*) spectra of the molecule reveal molecular resonances with onsets at −2.2 V and +0.8 V (marked by arrows in Figure 1B), corresponding to positive and negative ion resonances of PtPc. These resonances represent tunneling channels through the highest occupied (HOMO) and the degenerate lowest unoccupied molecular orbitals (LUMO and LUMO + 1), respectively. The apparent transport gap of ∼3.1 eV observed for PtPc/4-ML NaCl/Ag(111) agrees with prior experimental studies^33,44–46^ and theoretical simulations (vertical gray lines in Figure 1B) performed using the Pauli master equation methods combined with first-principles calculations based on the density functional theory (DFT) (see Supporting Information 1.3 and 1.4). Constant height d*I*/d*V* maps (insets in Figure 1B) image the molecular frontier orbitals.

**Figure 1:**
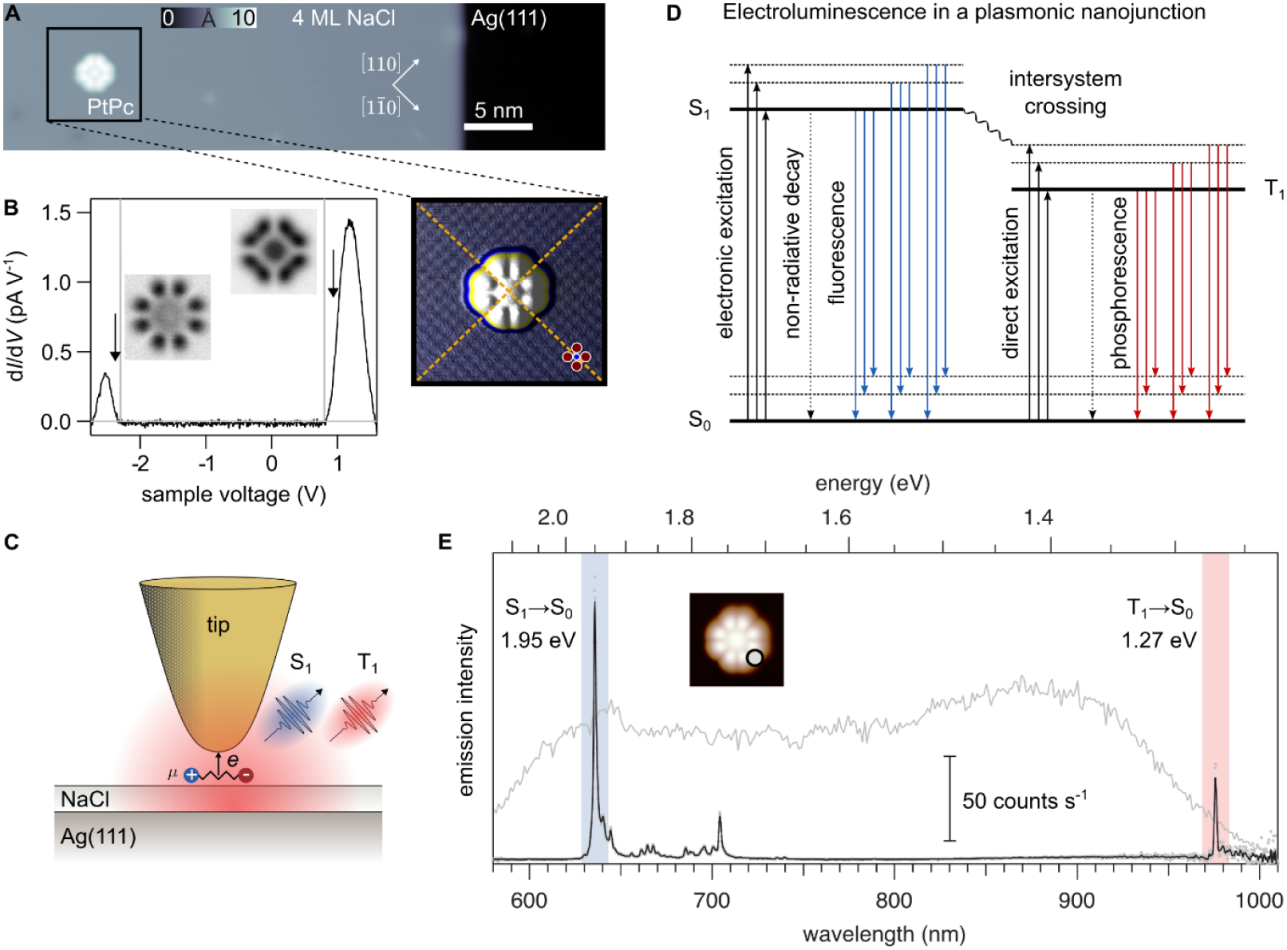
STML of a PtPc molecule. (A) Topography of a PtPc molecule adsorbed atop 4 ML NaCl/Ag(111) (*I* = 3 pA, *V* = 1 V). The inset shows a topograph (*I* = 3 pA, *V* = −2.6 V) with a color scale pronouncing the NaCl lattice corrugation overlaid with simulated illumination from the left-hand side, emphasizing the molecular features. (B) d*I*/d*V* spectrum measured at the center of the molecule on 4 ML NaCl with positive (HOMO) and negative (LUMO) ion resonances and onsets indicated by arrows at *V* = −2.3 V and *V* = 0.9 V, respectively. Gray lines mark the HOMO and LUMO energies computed by DFT. Insets: Inverted contrast d*I*/d*V* constant height maps at *V* = −2.4 V (HOMO) and *V* = 0.95 V (LUMO) (size: 3.5 × 3.5 nm^2^, *V*_mod_ = 50 mV). Arrows in the spectrum on the left mark the voltages at which these maps are recorded. (C) Schematic of the experiment with a single PtPc atop NaCl/Ag(111). Excitonic emission due to charge recombination in the molecule is detected outside of the UHV chamber. (D) Perrin–Jabłoński diagram of molecular emission processes in a tip–sample nanocavity with competing vibronic relaxation and radiation rates. (E) STML spectrum of a single PtPc molecule showing S_1_ → S_0_ (blue) and T_1_ → S_0_ (red) emission lines with their vibronic satellites (set point: *I* = 80 pA, *V* = −2.6 V, integration time *t* = 60 s, grating: 300 grooves mm^–1^). The spectrum has been normalized by a pure plasmonic spectrum, shown in gray (*I* = 250 pA, *V* = −2.6 V, *t* = 1 s) recorded atop the NaCl layer at some distance from the molecule. The normalization accounts for both, spectral enhancement variations and wavelength-dependent detector sensitivity.

Figure 1C illustrates the STML experimental setup (see Figure S2 for the optical setup, including the extension for TEPL). Figure 1D presents a Perrin-Jabłoński diagram detailing molecular emission processes triggered by electronic excitation through charge injection into the frontier molecular orbitals. This results in the direct excitation of both S_1_ and T_1_ states, responsible for fluorescence and phosphorescence emission, respectively.

An STML spectrum for a single PtPc molecule, acquired at a negative sample voltage (*V* = −2.6 V), with the tip placed on the edge of the PtPc molecule (gray dot in the inset), is shown in Figure 1E. Sharp emission lines are observed at photon energies between 1.95 and 1.70 eV, and around 1.27 eV. The line at 1.95 eV is attributed to the electronic transition from the excited S_1_ state to the S_0_ ground state, as confirmed by prior STML^33,45,46^ and photoluminescence measurements,^41^ as well as time-dependent DFT (TD-DFT) calculations (see Table S3). Based on DFT calculations and Raman spectra of PtPc powder (see Figure S3), we attribute the peaks at energies between 1.94 and 1.75 eV to vibronic satellites.

Previous studies of PtPc in single crystal form,^36^ in solution,^37,39^ and in OLED devices^38^ report phosphorescence energies between 1.28 and 1.31 eV. Accordingly, the sharp emission line at 1.27 eV is assigned to phosphorescence emission by radiative transition from the excited T_1_ state to the S_0_ ground state. Another emission line at 1.38 eV, rarely observed for negative sample voltages (see Figure S4), coincides with a lifting of LUMO and LUMO + 1 degeneracy, likely due to a defect in the NaCl lattice beneath the molecule. This emission line is attributed to luminescence from the cationic species PtPc^+^, resembling the experimental conditions for cationic emission observed for free-base phthalocyanine.^47^ No excitonic emission is observed under positive sample voltages, despite earlier reports of fluorescence of the anionic species at 1.36 eV in STML experiments on NaCl-covered Ag(100).^33^

We note that an earlier STML study of PtPc failed to observe the triplet emission line.^33^ This discrepancy likely arises from the actual faintness of the triplet line, the requirement of efficient decoupling from the substrate (at least 3 ML, ideally 4 ML of NaCl), and strong plasmonic enhancement preferably extending far into the red wavelength range. Our findings confirm the absence of the cationic emission, which we observe sporadically only on defected adsorption sites, as reported above. The allowed doublet emission D_1_^+^ → D_0_^+^ of the cation requires a two-step excitation, in which the second step, T_1_ → D_*n*_^+^, appears to be not yet activated or at least inefficient at the applied voltages, as illustrated by the energy scheme of Figure 2E. Alternatively, a direct excitation from the S_0_ ground state to the D_1_^+^ state would be possible. However, that excitation path requires a bias voltage below −3.58 V (Figure 2E) which is not reached in our experiments.

**Figure 2:**
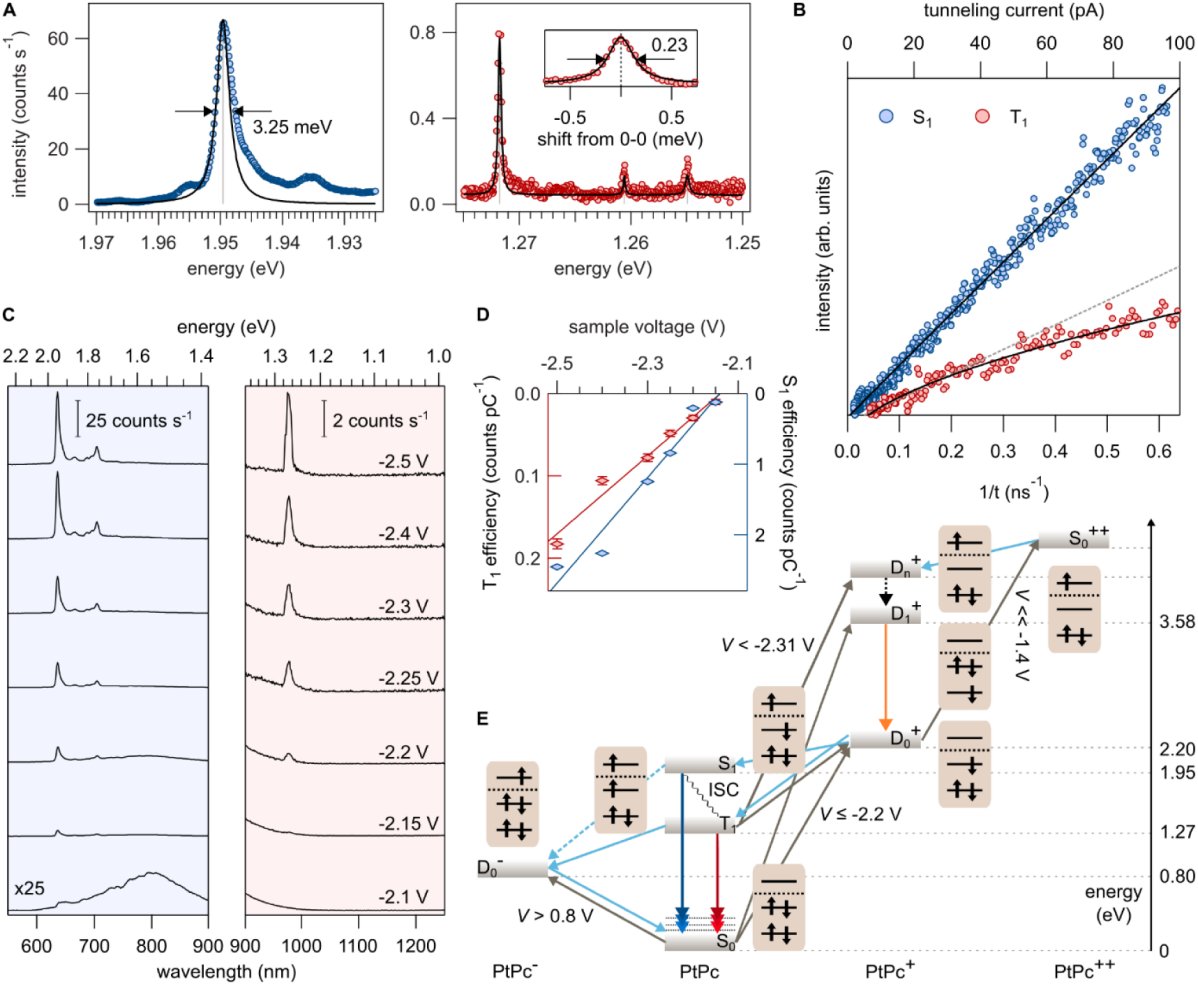
Electronic characterization of molecular emission. (A) Lorentzian line shape (black) fit of the S_1_ (left) and T_1_ (right) emission line and full-width at half-maximum (FWHM). The gray lines mark the peak position of the Lorentzian. Inset (right). Blowup of the T_1_ emission line with respect to peak position (1.272 eV) (*I* = 80 pA, *V* = −2.6 V; for S_1_: *t* = 5 min; for T_1_: *t* = 50 min, grating: 1200 grooves mm^–1^). (B) Current dependence of fluorescence and phosphorescence intensity at fixed voltage *V* = −2.6 V (for S_1_: *t* = 200 ms, for T_1_: *t* = 1 s). The S_1_ data is best fitted by *I* ^0.99^; the T_1_ data is fitted using a three-state model.^63^ The dashed gray line is a linear fit for the T_1_ data in the range: 0 pA – 40 pA. (C) S_1_ and T_1_ spectra at the sample voltages indicated (*I* = 200 pA, *t* = 30 s, grating: 50 grooves mm^–1^). The plasmonic spectrum (bottom) is measured at sample voltage *V* = −2.1 V and is multiplied by 25 for clarity (*t* = 1 s). (D) Integrated peak intensities from Lorentzian fits to the spectra in C. The linear extrapolation (solid lines) yields a cutoff voltage of *V* = −2.15 V. (E) Energy diagram of accessible molecular states for 4 different charge states of PtPc.

We performed TD-DFT calculations (see Supporting Information 1.2 and 1.3) for comparison with our experimental observations. These calculations yield S_1_ and T_1_ exciton energies of 2.02 and 1.03 eV, respectively, in fair agreement with the observed peaks at 1.95 and 1.27 eV. The calculated intensity ratio of the transition dipole moments $\mu_{T_{1}\to S_{0}}:\mu_{S_{1}\to S_{0}}$ is ∼1:40 (see Supporting Information 1.3). Experimentally, we find that the T_1_:S_1_ intensity ratios range between ∼1:3 (Figure 1E) and ∼1:10 (Figure 4C) after correcting for the wavelength dependencies of the plasmonic enhancement and the detector sensitivity.

Next, we discuss the excited-state lifetimes τ derived from the observed line width using the uncertainty principle (Δ*E*·Δτ ≥ ℏ). It is known that STML emission peaks may include multiple transitions and be broadened by multiple anti-Kasha transitions.^12,13^ Nevertheless, a longer T_1_ lifetime would always result in sharper emission lines when controlling for other parameters. Figure 2A shows fluorescence (left) and phosphorescence (right) spectra obtained using a high-resolution 1200 grooves mm^–1^ grating. A Lorentzian fit (black line in Figure 2A) yields a line width of 2.49 meV (τ ≥ 0.3 ps) for fluorescence and 0.23 meV (τ ≥ 2.9 ps) for phosphorescence. We use only the high-energy side of the lines to determine the full line width because the strong peaks are asymmetrically broadened to the lower energy side. The evaluation thus suggests a substantially longer lifetime for the emission at 1.27 eV with respect to the 1.95 eV line, supporting our assignment of the 1.27 eV peak to phosphorescence. Note, however, that the life times given above only provide lower limits.

In order to reveal the exciton formation mechanism in PtPc, we analyze the voltage and current dependence of the luminescence intensity (see Figure 2D and B, respectively). Both fluorescence and phosphorescence emissions onset simultaneously at a sample voltage of V ∼ −2.15 V, which is well above the singlet emission energy of 1.95 eV. The emission onset is thus not due to the quantum cutoff but coincides with the alignment of the tip Fermi energy with the edge of the positive ion resonance (−2.2 V, see Figure 1B) within experimental error. This indicates that the first step in both S_1_ and T_1_ excitation is a removal of an electron from the HOMO. Although the existence of excitation by an inelastic process cannot be ruled out for less negative voltage, no emission is observed below the onset of the positive ion resonance.

We performed theoretical calculations based on the Pauli master equation approach combined with the DFT and TD-DFT calculations to simulate STML for the PtPc molecule adsorbed on NaCl films grown on Ag(111). These calculations reproduce the positive and negative ion resonances at sample voltages of +0.75 V and −2.35 V, respectively, corroborating the simultaneous onset of fluorescence and phosphorescence at the HOMO onset. These findings confirm that the excitation occurs by charge injection via the lowest-lying molecular cationic state.^48^

Next, we analyze the fluorescence and phosphorescence intensities at −2.6 V sample voltage as a function of tunneling current (Figure 2B). We find a linear onset for both emission intensities, confirming a one-electron tunneling process via hole creation in the HOMO, as discussed above. An electron capture from the metal substrate by the LUMO is the second step, necessary to create the exciton, as illustrated in Figure 2E. This second charge transfer with the substrate restores molecular charge neutrality and thus preserves linearity in the tunneling current. However, at currents exceeding 80 pA, the phosphorescence intensity starts to deviate from linearity. At this current the time between tunneling electrons is still as long as ∼2 ns. This behavior can be attributed to the onset of saturation of triplet occupancy, allowing us to estimate an upper limit for the triplet lifetime to be ∼1 ns. The measured line width, however, indicates a lower bound for the triplet lifetime limit of ∼3 ps (as discussed above). It is worth noting that the early saturation of T_1_ state may be enhanced by an increased nonradiative decay rate as the metallic tip approaches the molecule with increasing tunneling current. Compared to the S_1_ state, the T_1_ state will generally be more susceptible to variations in nonradiative decay due to its inherently longer lifetime.

Figure 2E provides an overview of the energy levels of PtPc for the excitation and emission of the S_1_ and T_1_ states. Experimentally accessible energies are marked on the right-hand scale. As established earlier, the first step is the extraction of an electron from the HOMO, leading to the formation of a doublet state of the cationic species, D_0_^+^. Depending on the spin of the injected electron, this facilitates population of either the S_1_ or T_1_ state. When excitons are generated via charge-injection, a simple spin-statistics argument suggests a 3:1 ratio favoring T_1_ over S_1_ excitation due to the multiplicities of these states.^22^ However, this ratio may be slightly reduced because the tunneling barrier for triplet excitation is higher than that for singlet excitation.^34^

A longer lifetime of the triplet state relative to the singlet state further implies that the triplet state occupation will be on average higher than the singlet state occupation. Nevertheless, the contrasting experimentally observed low phosphorescence to fluorescence intensity ratio, ranging from ∼1:3 to ∼1:10 (see Figures 1E, 2C and 4C) can be explained by two other factors. First, the coupling strengths for radiative decay to the plasmon field likely differs between the two types of emission, despite both fluorescence and phosphorescence being enhanced by the localized plasmon field.^49–51^ To account for the pure wavelength dependence of this enhancement and detector sensitivity, the spectra have already been divided by the tip plasmonic spectrum. Second, the triplet state lifetime is long enough to let nonradiative decay channels dominate the decay. The possible decay of the triplet state T_1_ to the anionic D_0_^–^ and especially to the cationic D_0_^+^ state (see Figure 2E) through an “exciton ionization”-like process within the STM electric field will be very important and may best explain the absence of strong phosphorescence. This decay path may also explain why we do not observe a triplet lifetime in the range of 10–100 μs, as reported for the pentacene molecule by Peng et al.^52^ or 170–670 μs reported for PTCDA by Sellies et al.^32^ In these experiments, a spontaneous decay to a charged state is prohibited. In addition, the theoretical exciton decay rates obtained for the gas phase (see Methods) cannot serve as a realistic comparison for our experiment because the calculation assumes a perfect decoupling from the metallic substrate. We note that, under similar experimental conditions (few ML NaCl atop a metal substrate), Kaiser et al.^34^ derived triplet state lifetimes for ZnPc between 0.09 and 0.37 ns using a dynamic model.

To further assess the role of the cationic molecular state in emission, we return to the energy diagram in Figure 2E. The D_1_^+^ → D_0_^+^ transition requires a two-step charge injection process. The first step involves charge injection from the ground state S_0_ of the neutral molecule to D_0_^+^, which is reached at the HOMO onset energy. From D_0_^+^, a second excitation step to S_0_^2+^ can relax to the excited state D_1_^+^. This intermediate doubly charged state must overcome a strong intramolecular Coulomb repulsion. Alternatively, the D_0_^+^ state may first relax to the S_1_ or T_1_ states, from where a second charge injection step is needed to reach D_*n*_^+^. The second excitation step thus starts either from the S_1_ state, which decays efficiently by fluorescence, or from the T_1_ state, which can readily decay into the D_0_^–^ state. Although we cannot determine the precise energy of the D_*n*_^+^ state in Figure 2E, we may assume that the bias voltage necessary to reach it from T_1_ should lie below −2.2 V thus introducing a second threshold voltage close to the threshold of D_0_^+^ creation. Note that a direct excitation from T_1_ → D_1_^+^ is not possible in a single injection step. Most importantly, both of these routes would have a clear signature in the form of a superlinear tunneling current dependence. In contrast, the data in Figure 2B do not show such behavior. Based on this evidence and the observation of the 1.27 eV emission line in photoluminescence (see next section), we conclude that this emission cannot originate from a charged trionic state.

### STM Tip-Enhanced Photoluminescence (TEPL) of a Single Phosphor

Besides the direct excitation of the T_1_ state by charge injection, ISC from the S_1_ to the T_1_ state also contributes to phosphorescence.^22,42,53^ To investigate the role of ISC in single molecule phosphorescence in a controlled manner, we study PtPc using the recently demonstrated method of resonant TEPL^12–14^ with laser light resonantly exciting the S_1_ state, thereby maximizing molecular excitation. Figure 3A illustrates the experimental setup. A low-pass filter (LPF) blocks the excitation line, while a charge-coupled device (CCD) camera detects the phosphorescence. For excitation, the laser is tuned to the S_1_ absorption line to ensure that the T_1_ state is populated predominantly via ISC from the S_1_ state, with no contribution from indirect excitation of the T_1_ state via charged states as in STML (see Perrin–Jabłoński diagram in Figure 3B).

**Figure 3:**
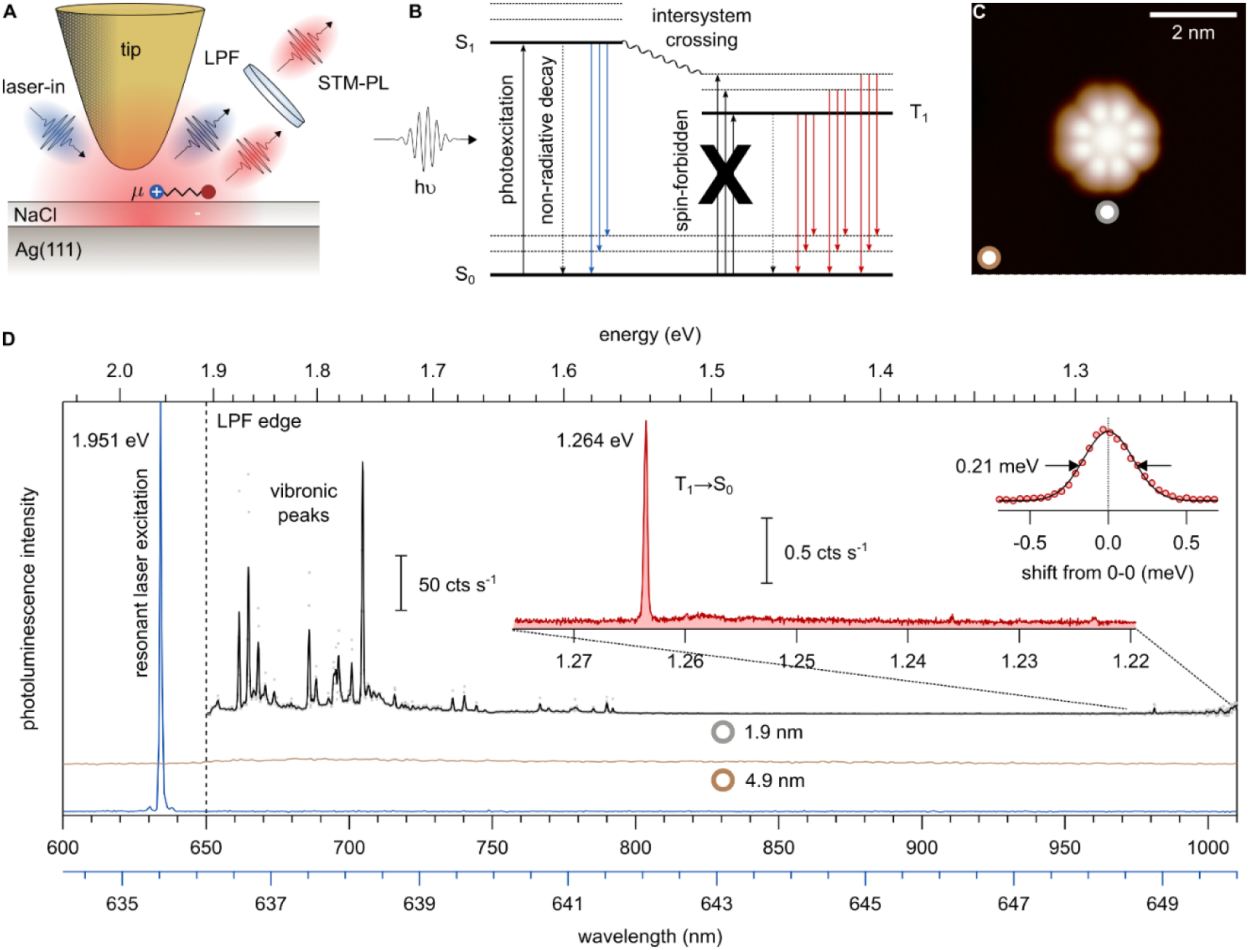
T_1_ emission in TEPL. (A) Schematic illustration of the TEPL experiment in which S_1_ is resonantly excited by incident laser light. (B) Schematic demonstrating that T_1_ can only be excited via intersystem crossing (ISC). (C) STM topography image of the PtPc molecule adsorbed atop 4 ML NaCl on Ag(111) (*I* = 3 pA, *V* = −2.6 V). (D) Laser line (blue, 635.52 nm, grating: 1800 grooves mm^–1^) used for resonant photoexcitation of the S_0_ → S_1_ transition. The laser spectrum is plotted with respect to the lower of the two wavelength scales. TEPL spectra measured at the positions indicated by the white dots with colored rings in (C). Distance from the center of the molecule: brown, *r* = 4.9 nm; gray, *r* = 1.9 nm (laser power: 1 μW, *t* = 60 s, grating: 300 grooves mm^–1^). The dashed line marks the 650 nm edge of the long-pass filter LPF. Inset: Zoom-in on the phosphorescence region of the TEPL spectrum (*I* = 3 pA, *V* = 1 V; laser power: 1 μW, *t* = 60 min, grating: 1200 grooves mm^–1^). The second inset shows the detailed T_1_ → S_0_ transition and a Lorentzian fit of the T_1_ line with the indicated FWHM.

The TEPL data (Figure 3D) is recorded at a low tunneling current (*I* = 3 pA) and a sample voltage of *V* = 1 V which excludes any plasmon-mediated optical excitation of the molecule. Moreover, the STM tip is positioned at a lateral distance of 1.9 nm from the molecule’s center (as indicated by the gray ring in Figure 3C) to exclude any electron injection into the molecule. The observed STM-PL spectrum reveals vibronic satellites of the S_1_ peaks down to 1.6 eV and T_1_ emission at 1.264 eV, in close agreement with the STML spectrum in Figure 1E (see also Figure S3). To the best of our knowledge, this is the first direct observation of ISC in a single-molecule STM experiment. A slight shift (∼8 meV) in phosphorescence between STML (1.272 eV, *V* = −2.6 V) and TEPL (1.264 eV, *V* = 1 V) is observed, which can be attributed to tip-induced effects, like the Stark shift as the measurements are performed with different applied voltages^9,27,54,55^ (see schematic in Figure 4A). Additional measurements of the Stark effect in T_1_ emission are shown in Figures S6 and S7.

**Figure 4:**
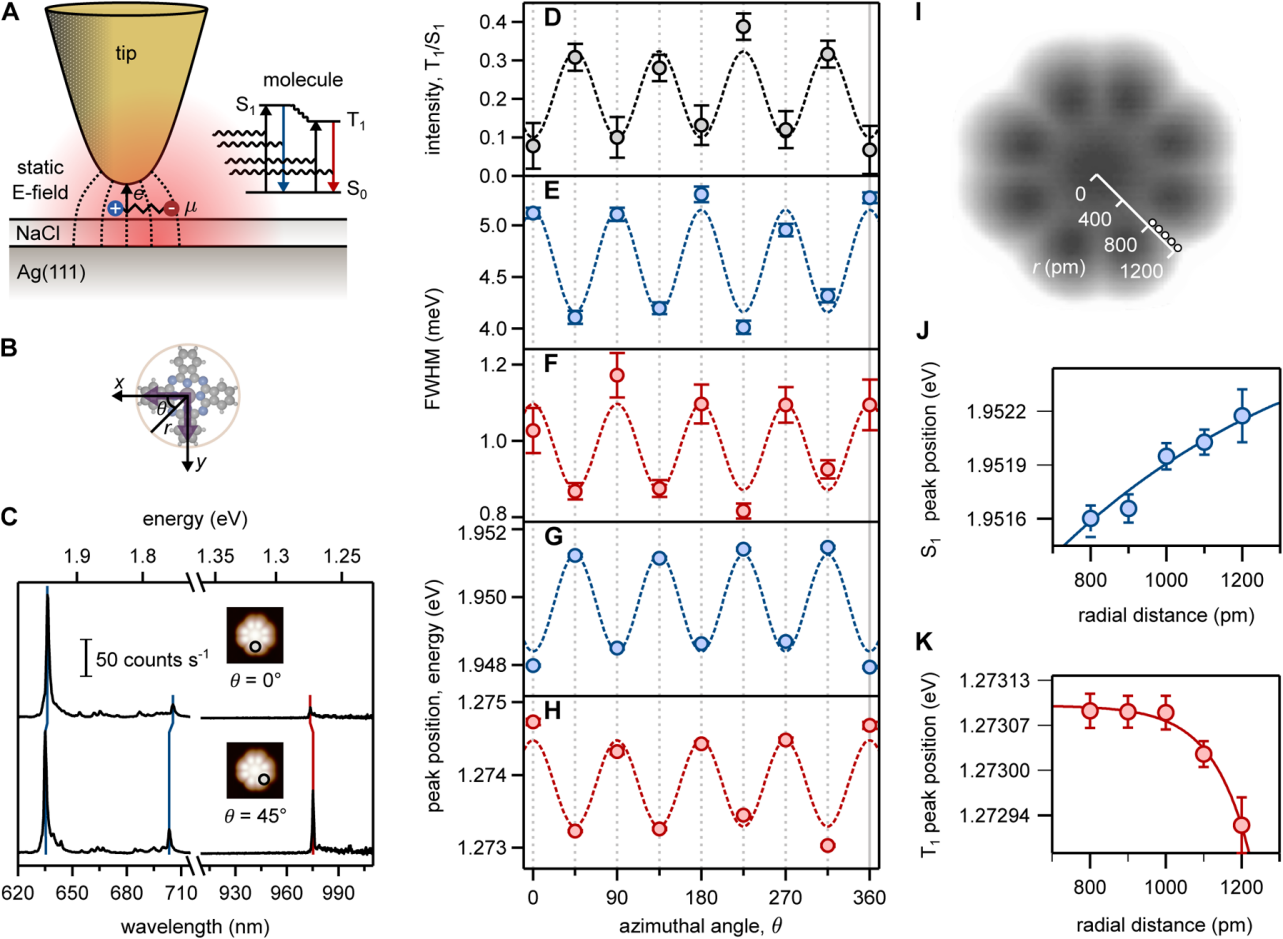
Azimuthal and distance dependence of S_1_ and T_1_ emission. (A) Illustration of the interaction between the plasmonic field in the nanocavity and the molecular fluorescence and phosphorescence together with the tip-induced electric field gradient over the molecule (red). (B) Schematic defining the polar coordinates *r* and θ with respect to the molecular geometry. (C) STML spectra obtained for θ = 0° and 45° (*r* = 1.1 nm; *I* = 50 pA, *V* = −2.6 V, for S_1_: *t* = 5 s and for T_1_: *t* = 30 s; grating: 300 grooves mm^–1^). (D) Azimuthal dependence of the T_1_ intensity normalized to the S_1_ intensity. The intensities are obtained as peak areas under Lorentzian fits. Error bars represent standard deviation of the peak area in the fit. (E, F) Azimuthal dependence of S_1_ (blue) and T_1_ (red) peak widths. (G, H) Azimuthal dependence of peak position of S_1_ and T_1_ emission lines obtained from Lorentzian fits. (I) STM topography image of a PtPc molecule with tip distance scale (*I* = 4 pA, *V* = −2.6 V). (J, K) S_1_ and T_1_ peak energies as a function of radial distance from the center of the molecule. The solid lines are exponential fits to guide the eye. Intensity ratio and line widths as a function of the radial distance for this data series are shown in Figure S8.

The line width of the phosphorescence in TEPL (0.21 meV, inset in Figure 3D) is comparable to that in STML (0.23 meV, right panel in Figure 2A), suggesting that the lifetime of the T_1_ state does not differ significantly between the two excitation methods. Combined with the saturation of phosphorescence with increasing tunneling current (Figure 2B), this finding affirms that the lifetime of T_1_ state is comparable to or longer than the vibrational relaxation time.

It is important to note that the intensity ratio between phosphorescence and fluorescence is not directly accessible in resonant TEPL. Since the S_0_ → S_1_ transition is used for excitation, a strong background intensity is present on the fluorescence line, which must be suppressed using an LPF. Thus, we use the S_1_ vibronic satellites present in both STML (Figure 1D) and TEPL (Figure 3D) to compare the phosphorescence intensities for both excitation methods. The ratio of phosphorescence intensity to the major S_1_ vibronic peaks is approximately 2 orders of magnitude lower in TEPL than in STML. The phosphorescence intensity observed in TEPL directly provides a lower bound for the ISC contribution in STML and constitutes a clear observation of the occurrence of ISC in an STM experiment at the single-molecule level.

### Intersystem Crossing and Exciton–Plasmon Coupling

In order to estimate the ISC rate, we analyzed the STML and TEPL spectra under the assumption that the intramolecular dynamics is similar in both experiments. By comparing the T_1_:S_1_ intensity ratios, we find that the ISC rate is about one tenth of the singlet decay rate. For the detailed calculation and the approximations used, see Supporting Information 1.4 and Figure S1. Based on the observed fluorescence line width (as discussed above), the ISC rate is coarsely estimated at ∼0.3 ps^–1^. This value aligns well with literature value (0.67 ps^–1^) of Pd-Phthalocyanine in α-chloronaphtalene at 4 K.^37^

While assessing the ISC rate required the assumption that electroluminescence and photoluminescence follow similar intramolecular dynamics, detailed investigations reveal deviations when the tip position relative to the molecule varies. In order to quantify the effects induced by plasmonic coupling, we investigate spectra recorded at varying tip position. An overview of the angular dependence of STML and TEPL data are presented in Figure S5.

First, we record constant current STML spectra with the tip placed at different positions on a circular path around the molecule’s center (Figure 4B) at a radius *r* = 1.1 nm and azimuths θ = *n*·45°, where *n* is an integer. The results indicate that the T_1_:S_1_ intensity ratio is significantly higher at θ = 45° (Figure 4C), demonstrating the critical role of the position of the plasmonic tip. The charge injection process, which is similar for the generation of both excitons, would not explain a dependence on θ. Figure 4D shows that the T_1_:S_1_ intensity ratio is generally higher at azimuthal angles θ = (2*n* + 1)·45° and lower at θ = *n*·90°. The ratio is in antiphase with the line widths of both fluorescence and phosphorescence (Figure 4E,F), which broaden with increased X–P coupling strength.^2,9,40,56,57^

Specifically, the S_1_ line width variation of ∼22% (Figure 4E) indicates a shortening of the S_1_ lifetime at θ = *n*·90°, while the T_1_ line width (Figure 4F) variation of ∼29% is in-phase with the S_1_ line width broadening. In contrast to the X–P coupling, it is reasonable to assume that the ISC rate is not significantly affected by the tip position, as ISC is primarily induced by the Pt atom at the molecular center^42^ rather than by the atoms of the tip. The lifetime shortening observed is therefore mainly attributed to enhanced X–P coupling, which accelerates the radiative rate from S_1_, thereby reducing the branching ratio of the competing ISC. Since ISC converts S_1_ to T_1_, it directly alters the T_1_:S_1_ intensity ratio. Therefore, the variation observed in Figure 4D is considered to reflect changes in the ISC branching ratio induced by changes in X–P coupling. We remark that a concomitant change of the radiative-to-nonradiative branching ratio of the T_1_ state itself could also affect a detailed quantitative evaluation. The tip position dependency of the T_1_:S_1_ ratio provides a means to control the weight between current-induced fluorescence and phosphorescence, thereby enabling emission color tuning in plasmonic nanocavities.

In Figure 4G, the S_1_ peak energy at azimuthal positions θ = (2*n* + 1)·45° is blueshifted compared to θ = *n*·90°. This observation is consistent with earlier studies reporting Lamb effect-induced redshift of the S_1_ emission line.^9,11,12,57^ However, we find that the energy shift of the phosphorescence line as a function of azimuth (Figure 4H) is opposite to the one of the fluorescence line. A similar opposite behavior between fluorescence (Figure 4J) and phosphorescence (Figure 4K) is observed when the tip is moved away from the center of the molecule (Figure 4I). These suggest competing Lamb and Stark effects, acting in opposite directions but with different amplitudes for the two transitions. Such behavior has been reported previously,^9^ but a quantitative disentanglement of the contributions would require further investigation.

## Conclusions

This study provides a detailed picture of nanoscale processes in plasmon-enhanced phosphorescence. The unique combination of STML and TEPL facilitates the analysis of phosphorescence and allows a direct observation of singlet–triplet ISC of excitons at the single-molecule scale. The study demonstrates that coupling to a plasmonic cavity significantly affects the intensities of the singlet and triplet excitons of an isolated PtPc molecule. For the used NaCl/Ag(111) substrate, both excitons are observed in STML only under negative polarity, with their onset at the alignment of the HOMO with the tip Fermi energy. The findings indicates that phosphorescence—when corrected for plasmonic enhancement by the STM tip—even though observable, remains much weaker than fluorescence due to more efficient nonradiative decay pathways for the triplet compared to the singlet. The coupling to a plasmonic structure shortens the singlet lifetime, increases fluorescence intensity, reduces triplet occupation via ISC, and consequently results in lower phosphorescence intensity. These results suggest that X–P coupling could be used to modulate fluorescence and phosphorescence in OLED applications.^27^ Future studies could explore phosphors whose triplet and singlet transitions are closer to each other in energy, allowing for an increased ISC. Moreover, suppressing the decay of the triplet state to anionic or cationic molecular states may significantly improve phosphorescence efficiency. The experimental insights presented here have implications for plasmonic OLED devices,^27,58^ bioimaging,^59,60^ and biosensing,^61,62^ where exciton dynamics play a central role.

## Methods/Experimental

### Sample Preparation

An Ag(111) single-crystal is prepared by repeated sputter-annealing cycles. NaCl is then evaporated thermally from a Knudsen cell on the crystal surface. Using a homemade evaporator, PtPc is finally deposited onto the NaCl-covered Ag(111) directly in the STM head held at 4.5–10 K. For further details, see Supporting Information 1.1.

### DFT Analysis

Electronic and vibrational structures of PtPc are analyzed using first-principles calculations based on density functional theory (DFT) and time-dependent DFT (TD-DFT) in order to simulate molecular luminescence spectra. Furthermore, optical transition rates are evaluated for the gas phase molecule. A description of these calculations including their detailed results are presented in Supporting Information 1.2 and 1.3.

## Supplementary Material



## Notes

Sample preparation and details of STM measurements; DFT and TD-DFT analysis of the molecular luminescence spectra including vibronic transitions and transition energies between molecular many-body states; DFT and TD-DFT analysis of the optical transition rates for the gas phase molecule; estimation of the intersystem crossing rate; model for fluorescence and phosphorescence; experimental setup; comparison of vibronic satellites for fluorescence and phosphorescence; emission from charged PtPc molecule; angle dependence of fluorescence and phosphorescence; extended TEPL spectra at different voltage polarities demonstrating the Stark shift of T_1_ emission; voltage dependence of the T_1_ emission line comparing STML and TEPL; radial distance-dependent X–P coupling (Purcell effect) of the S_1_ and T_1_ emissions (PDF)

## Abbreviations

STM: scanning tunneling microscopy
UCEL: electroluminescence upconversion
ISC: intersystem crossing
SOC: spin–orbit coupling
OLED: organic light emitting diode
PtPc: platinum­(II) phthalocyanine
UHV: ultrahigh vacuum
ML: monolayer
X-P: exciton–plasmon
HOMO: highest occupied molecular orbital
LUMO: lowest unoccupied molecular orbital
STML: STM-induced electroluminescence
TEPL: tip-enhanced photoluminescence
DFT: density functional theory
LPF: low-pass filter
CCD: charge-coupled device
