# Supplementary material for: Single-Molecule Phosphorescence and Intersystem Crossing in a Coupled Exciton Plasmon System
Source: ACS Nano. 2025 Jun 25;19(26):23796–805. doi: 10.1021/acsnano.5c04193 (PMC12257640; doi:10.1021/acsnano.5c04193)
Supplement: Supplementary file 1 [file nn5c04193_si_001.pdf]

Supporting information

Single-molecule phosphorescence and intersystem crossing in a coupled exciton-plasmon system

Abhishek Grewal[1*], Hiroshi Imada[2*], Kuniyuki Miwa[3], Miyabi Imai-Imada[2], Kensuke Kimura[2], Rafael Jaculbia[2], Klaus Kuhnke[1*], Klaus Kern[1,4], and Yousoo Kim[2*]

[1]Max-Planck-Institut für Festkörperforschung, Heisenbergstrasse 1, Stuttgart 70569, Germany

[2]Surface and Interface Science Laboratory, RIKEN, Wako, Saitama 351-0198, Japan

[3]Institute for Molecular Science, Myodaiji, Okazaki, Aichi 444-8585, Japan

[4]Institut de Physique, École Polytechnique Fédérale de Lausanne, Lausanne 1015, Switzerland

1. Supporting text

1.1 Sample preparation and details of STM measurements

The Ag(111) single-crystal (> 99.999% purity) is cleaned by repeated cycles of Ar^+ ion sputtering and subsequent annealing. NaCl is then evaporated thermally from a Knudsen cell heated to 890 K on the Ag(111) surface held at room temperature. For these preparation conditions, defect-free (100)-terminated two, three, and four monolayers thick NaCl islands are obtained. PtPc is deposited onto the NaCl-covered Ag(111) directly in the STM head at 4.5 K - 10 K using a homemade evaporator heated to 638 K. An electrochemically etched gold wire[1] (99.95% purity) is used as tip and conditioned by controlled indentation and voltage pulses on the clean Ag(111) surface. For the experiment, it is crucial to have broad plasmonic resonances spanning the range from S_1 to T_1 emission of PtPc. Therefore, the plasmonic resonance of the tip-sample junction is characterized using STML. A typical resonance of the tip-sample cavity plasmon is shown in Fig. 1E (grey trace).

1.2 DFT and TD-DFT analysis of the molecular luminescence spectra including vibronic transitions and transition energies between molecular many-body states

Electronic and vibrational structures of PtPc were analyzed using first-principles calculations based on density functional theory (DFT) and time-dependent DFT (TD-DFT) implemented in the software package Gaussian 16.[2] All calculations were performed using the Ahlrichs triple-zeta valence basis set with polarization and diffuse functions (def2-TZVPD).[3, 4] Effective core potential was utilized to substitute the 60 core-orbitals of the Pt atom.[5] First, the geometry for the ground electronic state of the neutral molecule was optimized using the hybrid B3LYP functional.[6] Next, according to the tuning procedures,[7] the optimal value of the system-specific range separation parameter ω was determined as 0.151 Bohr^{-1} for a range-separated hybrid density functional (LC- wHPBE).[8] Hereafter, the tuned range-separated functional was referred as LC-wHPBE*. All subsequent calculations were performed at the LC-wHPBE*/def2-TZVPD level of theory.

The geometry optimizations were carried out for the ground electronic state of different charged molecules. The analysis of the vibrational frequencies for each charged molecule was performed to ensure that all positive frequencies were obtained. The simulations yield total energies $E_{N,a}$ of electronic many-body state $|N, a\rangle$, where N indicates the number of excess electrons with respect to the neutral molecule. The simulation results were shown in Supplementary Table X1. The results indicate the cationic, neutral, and anionic states ($N = -1, 0, 1$) can be accessed in the range of bias voltage used in the experiment. We thus focused on these charged states and excluded the doubly charged states ($N = \pm 2$).

Supporting Table X1 | The number of excess electrons N with respect to the neutral molecule, spin multiplicity s, total energy $E_{N,a}$ (sum of the electronic and zero-point vibrational energies), and energy difference from the total energy of $|0, S_0\rangle$.

Electronic state	N	s	$E_{N,a}$ (eV)	$\Delta E_{N,a}$ (eV)
$\vert 0, S_0\rangle$	0	singlet	−48579.831781	0
$\vert -1, D_0\rangle$	−1	doublet	−48573.441673	+6.390107
$\vert -2, S_0\rangle$	−2	singlet	−48563.839070	+15.992711
$\vert -2, T_0\rangle$	−2	triplet	−48563.018210	+16.813570
$\vert +1, D_0\rangle$	+1	doublet	−48582.017591	−2.185810
$\vert +2, T_0\rangle$	+2	triplet	−48581.101356	−1.269575
$\vert +2, S_0\rangle$	+2	singlet	−48581.119451	−1.287670

The vertical excitation energies for the neutral, cationic, and anionic states were calculated using the TDDFT at the LC-wHPBE*/ Def2TZVPPD level. Supporting Table X2 exhibits the corresponding energies and transition dipole moments. To analyze molecular luminescence spectra, geometry optimization and frequency analysis for the first excited electronic states with both singlet and triplet spin multiplicities were performed. The simulation results of the total energies were displayed in Supporting Table X3. The obtained information on the molecular vibrations was utilized to evaluate the vibrational overlap integrals $\langle v_{n'}^{N,a'} | v_n^{N,a} \rangle$ associated with the optical transition of the neutral molecules, using the method implemented in Gaussian 16.[9-12] The results are shown in Supporting Figure 2.

Image interaction energy for PtPc molecule adsorbed on a NaCl ultrathin film growth on a metal surface was evaluated using the dielectric model introduced by Barone *et al.*.[12] The electron and hole attachment energies for the adsorbed molecule are shifted from the values for a molecule in the gas phase, because the molecule is positioned near the metal substrate. It had been reported that the differences are mainly attributed to the image interaction with the metal substrate. Following the literature, the shift for

the electron/hole attachment energies was estimated as 0.769 eV. Transition energy $\varepsilon_{Na,Mb}$between molecular many-body states $|N,a\rangle$ and $|M,b\rangle$ is calculated as $\varepsilon_{Na,Mb} = E_{N,a} + E_{N,a}^{\mathrm{Imag}} - E_{M,b} - E_{M,b}^{\mathrm{Imag}}$, where $E_{N,a}^{\mathrm{Imag}}$ indicates the correction of the total energies of the molecule owing to the image charge effects. Calculation results were summarized in Supporting Table X4. As the work function for NaCl/Ag (111) can be estimated as 3.57 eV,[13] we consider the following molecular many-body states in the theoretical analysis: $|0, S_0\rangle$, $|0, S_1\rangle$, $|0, S_2\rangle$, $|0, T_1^{m=0,\pm1}\rangle$, $|0, T_2^{m=0,\pm1}\rangle$, $\left|-1,\ D_0^{\sigma=\pm1/2}\right\rangle$, $\left|+1,\ D_0^{\sigma=\pm1/2}\right\rangle$, and $\left|+1,\ D_1^{\sigma=\pm1/2}\right\rangle$. It is noteworthy that $|0, S_1\rangle$, $|0, T_1^m\rangle$, and $|+1, D_0^\sigma\rangle$ are, respectively, energetically degenerated with $|0, S_2\rangle$, $|0, T_2^m\rangle$, and $|+1, D_1^\sigma\rangle$.

Supporting Table X2 | Vertical excitation energies E_{ex} from the ground electronic state to several lowest excited states and transition dipole moment μ for the neutral ($N = 0$), cationic ($N = -1$), and anionic states ($N = +1$) of the molecule. For the neutral molecule, the singlet spin multiplicity was accounted.

	$N = 0$		$N = -1$		$N = +1$	
	E_{ex} (eV)	μ (a.u.)	E_{ex} (eV)	μ (a.u.)	E_{ex} (eV)	μ (a.u.)
1	2.1591	2.7950	1.1699	0.3493	0.3532	0.0000
2	2.1591	2.7950	1.1699	0.3499	1.4666	0.6077
3	2.6076	0.0000	1.6838	0.0000	1.5546	0.0407
4	2.6985	0.0000	1.8407	1.2856	2.0013	1.0420
5	2.7931	0.0000	1.8407	1.2866	2.0629	2.9645

Supporting Table X3 | Total energy $E_{N,a}$ (sum of the electronic and zero-point vibrational energies) for singlet and triplet excited electronic states and energy difference from the total energy of $|0, S_0\rangle$.

Electronic state	$E_{N,a}$ (eV)	$\Delta E_{N,a}$ (eV)
$|0, S_1\rangle$	-48577.571532	2.019167
$|0, T_1\rangle$	-48578.557700	1.032999

Supporting Table X4 | Transition energy $\varepsilon_{Na,Mb}$between molecular many-body states $|N,a\rangle$ and $|M,b\rangle$.

$|N,a\rangle$	$|M,b\rangle$	$\varepsilon_{Na,Mb}$ (eV)
$|0, S_0\rangle$	$|-1, D_0^\sigma\rangle$	-5.6221
$|0, S_1\rangle$	$|-1, D_0^\sigma\rangle$	-3.6029
$|0, T_1^m\rangle$	$|-1, D_0^\sigma\rangle$	-4.5891
$|+1, D_0^\sigma\rangle$	$|0, S_0\rangle$	-2.9549
$|+1, D_0^\sigma\rangle$	$|0, S_1\rangle$	-4.9740
$|+1, D_0^\sigma\rangle$	$|0, T_1^m\rangle$	-3.9879

1.3 DFT and TD-DFT analysis of the optical transition rates for the gas phase molecule

Optical transition rates were evaluated with the DFT and TDDFT calculations implemented in the ORCA software package.[14, 15] As the spin-orbit coupling (SOC) should be included to evaluate the phosphorescence and ISC rates, we utilized this software package. Molecular structure for the first excited electronic state with the singlet spin multiplicity (S_1 state) was optimized at the B3LYP/def2-TZVP level

of theory. To accelerate the calculation, the resolution of identity approximation for the Coulomb part (RIJ) and the chain of sphere algorithm for the exchange part (COSX) were employed with the corresponding auxiliary basis sets.[16-19]

The relativistic effects were included by employing the zeroth-order regular approximation (ZORA) method.[20] The B3LYP functional was utilized with the scalar relativistic (SR) contracted version of the Ahlrichs triple-zeta valence basis set with polarization functions (ZORA-def2-TZVP) for H, C, and N atoms, and the segmented all-electron relativistically contracted (SARC-ZORA-TZVP) basis set for Pt atom.[2] The RIJCOSX approximation with auxiliary basis sets (SARC/J and def2-TZVP/C) was utilized to accelerate the calculations.[16-19, 21-25] Molecular geometry with the T_1 state was optimized with the SR ZORA Hamiltonian. Then, at the optimized geometry, the SOC was included as a perturbation into the SR ZORA calculation results, where 25 singlet and 25 triplet excited states were used as the basis for the perturbation expansion, to find the spin-mixed states and the finite amount of the transition dipole moments for the triplet-origin states.[26] According to the previous study,[27] the spin-orbit integrals were calculated with the RI-SOMF(1X) approximation[28] and TDDFT calculations were performed without Tamm-Dancoff approximation (TDA).[29]

The transition dipole moment $\mu_{\mathrm{S}_0-\mathrm{S}_1}$ for the $S_0 - S_1$ transition was evaluated at the optimized structure for the S_1 state and was obtained as 2.93 (a.u.). For the $\mathrm{S}_0 - \mathrm{T}_1$ transition, $\mu_{\mathrm{S}_0-\mathrm{T}_1}$ = 0.0686 (a.u.) was obtained at the optimized structure for the T_1 state. The fluorescence and phosphorescence rates were estimated using the formula $k = \frac{4}{3\hbar}\left(\frac{\omega}{c}\right)^3 |\mu|^2$ with $\hbar\omega$ being the optical transition energy, $\hbar$ the Planck constant divided by 2π, c the speed of light in vacuum, and μ the transition dipole moment.[30] The rate $k_{\mathrm{S}_0-\mathrm{S}_1} = 5 \times 10^7$ s^{-1} and $k_{\mathrm{S}_0-\mathrm{T}_1} = 5 \times 10^3$ s^{-1} were, respectively, utilized for the fluorescence and phosphorescence processes in the gas phase, that are at the same order of magnitude as calculated with the above-shown values of $\mu_{S_0-S_1}$ and $\mu_{S_0-T_1}$.

1.4 Estimation of the intersystem crossing rate

Supporting Figure 1 shows an oversimplified energy scheme (compare to Fig.2E) for electroluminescence of the studied PtPc molecule. In order to explore the steady state condition, we set up master equations for the singlet (n_s) and triplet (n_T) occupation numbers as follows:

$$\dot{n}_S = I_{el}\,\beta - n_S\,\frac{\tau'_S + \tau_{ISC}}{\tau'_S\ \tau_{ISC}} \xrightarrow{equilib} 0$$
$$\dot{n}_T = I_{el}(1-\beta) + n_S\,\frac{1}{\tau_{ISC}} - n_T\,\frac{1}{\tau'_T} \xrightarrow{equilib} 0 \qquad \textbf{(eqns. 1)}$$

Here τ_{ISC} is the inverse intersystem crossing rate, τ'_S the effective singlet state lifetime (composed of non-radiative τ_{nrS} and radiative τ_{rS} singlet lifetimes) and τ'_T the effective triplet state lifetime (see also Supporting Figure 1). Eqns. 1 describe the excitation by a tunnel current I_{el}. The spin multiplicity of triplet and singlet excitation is assumed to be 3:1 providing an excitation branching of $\beta = 0.25$ to the singlet state and $1 - \beta = 0.75$ to the triplet state. Both time derivatives tend towards zero when equilibrium is reached.

$$\dot{n}_S = I_{photo} - n_S\,\frac{\tau'_S + \tau_{ISC}}{\tau'_S\ \tau_{ISC}} \xrightarrow{equilib} 0$$
$$\dot{n}_T = n_S\,\frac{1}{\tau_{ISC}} - n_T\,\frac{1}{\tau'_T} \xrightarrow{equilib} 0 \qquad \textbf{(eqns. 2)}$$

describes the photo excitation where no direct excitation of the triplet state occurs, and I_{photo} is the excitation by light absorption.

In the following, we will make the coarse assumption that the internal rate constants of the model (Supporting Figure 1) are the same for phosphorescence and fluorescence and that the non-radiative decay of the S_1 state is negligible because the radiative decay dominates due to the Purcell effect in the STM.

Experimental observation of the steady state cannot access the time constants in the model directly, but allow to observe the triplet to singlet intensity ratios in electroluminescence $R_{el} = \frac{\eta_T}{\eta_S}\frac{n_{elT}}{n_{elS}}\frac{\tau_{rS}}{\tau_{rT}}$ and in

photoluminescence $R_{pl} = \frac{\eta_T}{\eta_S} \frac{n_{plT}}{n_{plS}} \frac{\tau_{rS}}{\tau_{rT}}$. Here η_S and η_T are the detection efficiencies for singlet and triplet emission.

Using these definitions together with eqns. 1, eqns. 2 and the above assumptions, we find that experimental detection efficiencies, the excitation strengths (I_{el} and I_{photon}) and the triplet state lifetime cancel out and we have:

$$\Delta := \frac{R_{el}}{R_{pl}} = \left(3 \frac{\tau_{ISC}}{\tau_{rS}} + 4 \right) \qquad (eq. 3)$$

From the experimentally observed triplet to singlet ratios discussed in the main text, we have $\Delta \approx 34$ and can derive:

$$\frac{\tau_{ISC}}{\tau_S} \approx \frac{\Delta - 4}{3} \approx 10 \qquad (eq. 4)$$

and

$$\frac{n_T}{n_S} \approx 10 \frac{\tau_S}{\tau_T} \qquad \text{(eq.5)}$$

Using the experimentally observed line width (2.49 meV) we obtain a coarse estimate of the singlet state life times of $\tau_S \approx 0.3$ ps which is of the same order as the literature value $\tau_S \approx 0.7$ ps derived by dynamic photon-correlation studies of Zn-Phthalocyanine.[31] We then find for the intersystem crossing $\tau_{ISC} \approx 3$ ps comparable to the experimental value for Pd-Phthalocyanine of $\tau_{ISC} \approx 1.5$ ps.[32]

2. Supporting figures

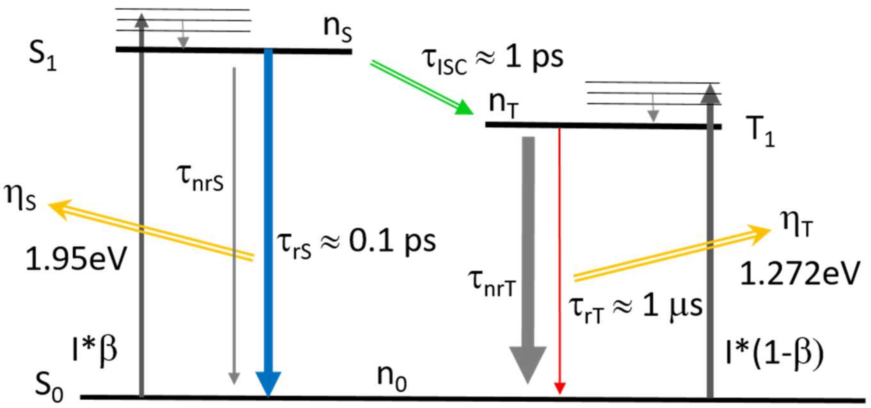

Supporting Figure 1. Model for fluorescence and phosphorescence introducing the variables used for the calculation. n_0 denotes the occupation of the S_0 ground state of the system. n_S and n_T denote the occupation of the lowest singlet (S_1) and lowest triplet (T_1) state, respecively. The life times, that are the inverse rate constants, are denoted by τ with the index denoting *nr* for non-resonant, *r* for resonant, and ISC for inter-system-crossing transitions. η_S and η_T are efficiency factors comprising the coupling of the respective transition to the far field, the intrinsic instrumental transmission and the detector efficiency. β controls the branching ratio between excitation of the singlet (β) and excitation of the triplet (1- β). The overall excitation efficiency of the system is also assumed to be included as a factor in the detection efficiencies η.

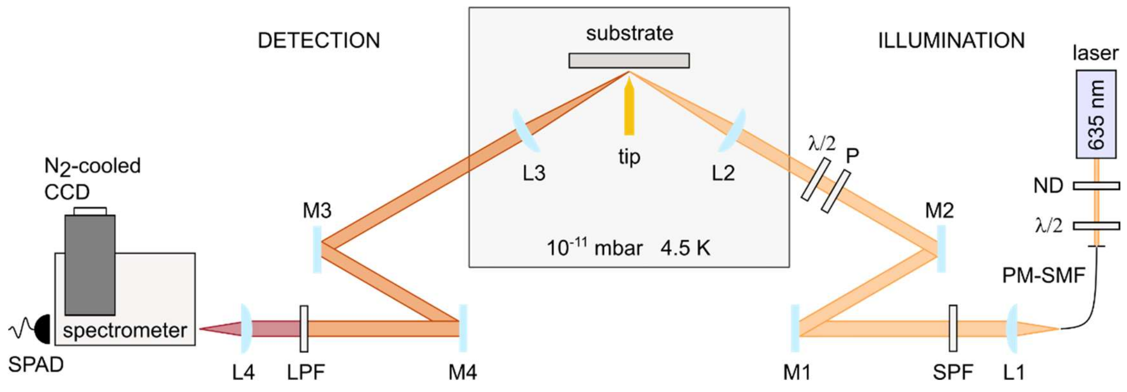

Supporting Figure 2. Experimental setup. Schematic illustration of the ultrahigh vacuum (UHV) low-temperature scanning tunneling microscope (Omicron) operating at 4.3 K. All experiments have been performed at the Surface and Interface Science Laboratory, RIKEN, Japan. The STM stage is equipped with two optical lenses (each covering a solid angle of ~ 0.5 sr). For the STML measurement, the emitted light was collimated by lens L3 and directed out of the UHV chamber, where it was refocused onto a grating (50, 300, or 1200 grooves mm^{-1}) spectrometer (Acton, SpectraPro 2300i) and detected with a charge coupled device (CCD) (Princeton, PyLoN:100) cooled with liquid nitrogen. A path switching mirror in the spectrometer allows to direct the light to an avalanche photo diode (APD: Excelitas SPCM-AQRH) which is used to obtain the current dependence of light emission. The pulses from the APD were counted using a multi-channel DAQ device (National Instruments) and monitored using a LabVIEW software package. For the TEPL measurement, excitation was induced using a laser diode (Thorlabs). Neutral-density filters (ND) were employed to control the laser power. The laser light was coupled to a polarization-maintaining single-mode fiber (PM-SMF: Thorlabs) and collimated by lens L1. Then the laser beam passes through a short pass filter (SPF: Semrock) to clean up the spectral region to be measured. Polarization is defined by a polarizer (p) and a half wave plate (λ/2). In this study, *p*-polarization is used. Finally, the laser is focused into the STM junction by lens L2. The emitted light is collimated by lens L3 and directed out of the UHV chamber, where it passes through a long pass filter (LPF: Semrock) to block the excitation laser light (the LPF is removed during STML measurements). The laser energy is tuned by controlling the temperature of the diode and monitored using a grating (1800 grooves mm^{-1}) spectrometer (Acton IsoPlane-320) with a nitrogen-cooled CCD photon detector (Princeton, Spec10).

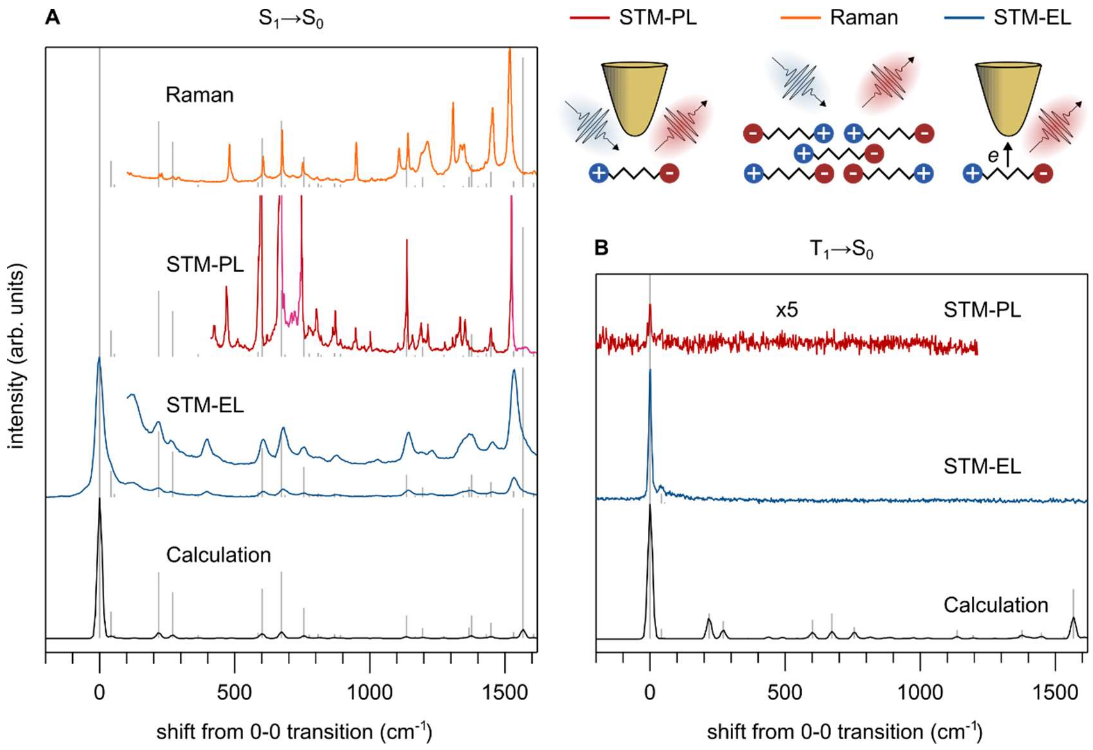

Supporting Figure 3. Comparison of vibronic satellites for fluorescence and phosphorescence. (A) Top to bottom: Raman spectrum for PtPc powder using a 532 nm laser (grating: 600 grooves mm^{-1}), TEPL spectrum next to a PtPc molecule atop 4 ML NaCl on Ag(111) (I = 3 pA, V = 1 V; laser power: 1 µW, t = 30 s, grating: 1200 grooves mm^{-1}), STML spectrum for the PtPc molecule obtained with the tip placed atop a HOMO lobe (I = 60 pA, V = −2.6 V; t = 120 s, grating: 300 grooves mm^{-1}), and calculated vibronic spectrum of a neutral PtPc molecule in the gas phase for the $S_1 \rightarrow S_0$ transition. (B) Top to bottom: TEPL (t = 120 s), STML, and calculated optical spectrum of a neutral PtPc molecule for the $T_1 \rightarrow S_0$ transition.

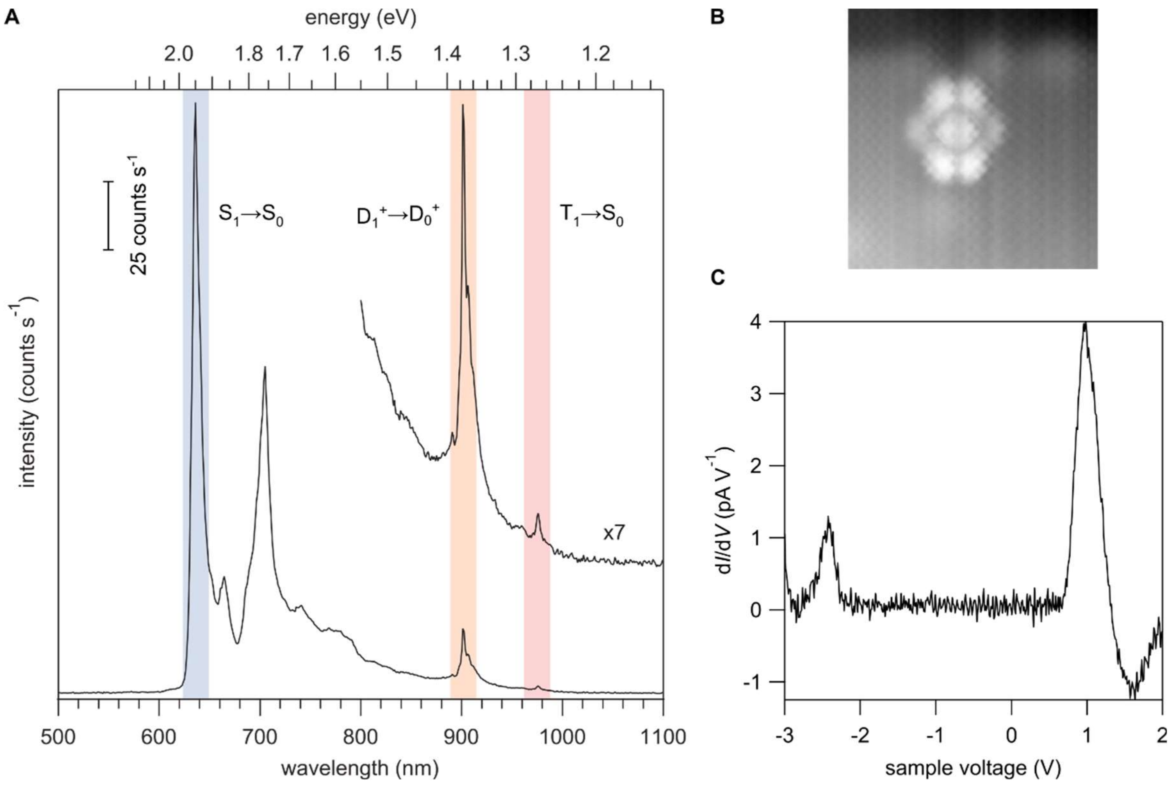

Supporting Figure 4. Emission from charged PtPc molecule. (A) STML spectrum obtained for a charged PtPc species ($PtPc^+$) adsorbed atop 3 ML NaCl on Ag(111) at negative sample voltage (I = 200 pA, V = -2.7 V, t = 200 s, grating: 50 grooves mm^{-1}). These STML spectra have not been normalized by the plasmon spectra in contrast to the other spectra of the study. (B) STM topography image of the molecule showing degeneracy lifting of the LUMO and LUMO+1 orbital (I = 4 pA, V = 1.1 V, size: 5×5 nm^2). (C) dI/dV spectrum obtained atop the center of the molecule showing the molecular frontier orbitals, similar to the case where only neutral emission is observed (see Fig. 1A).

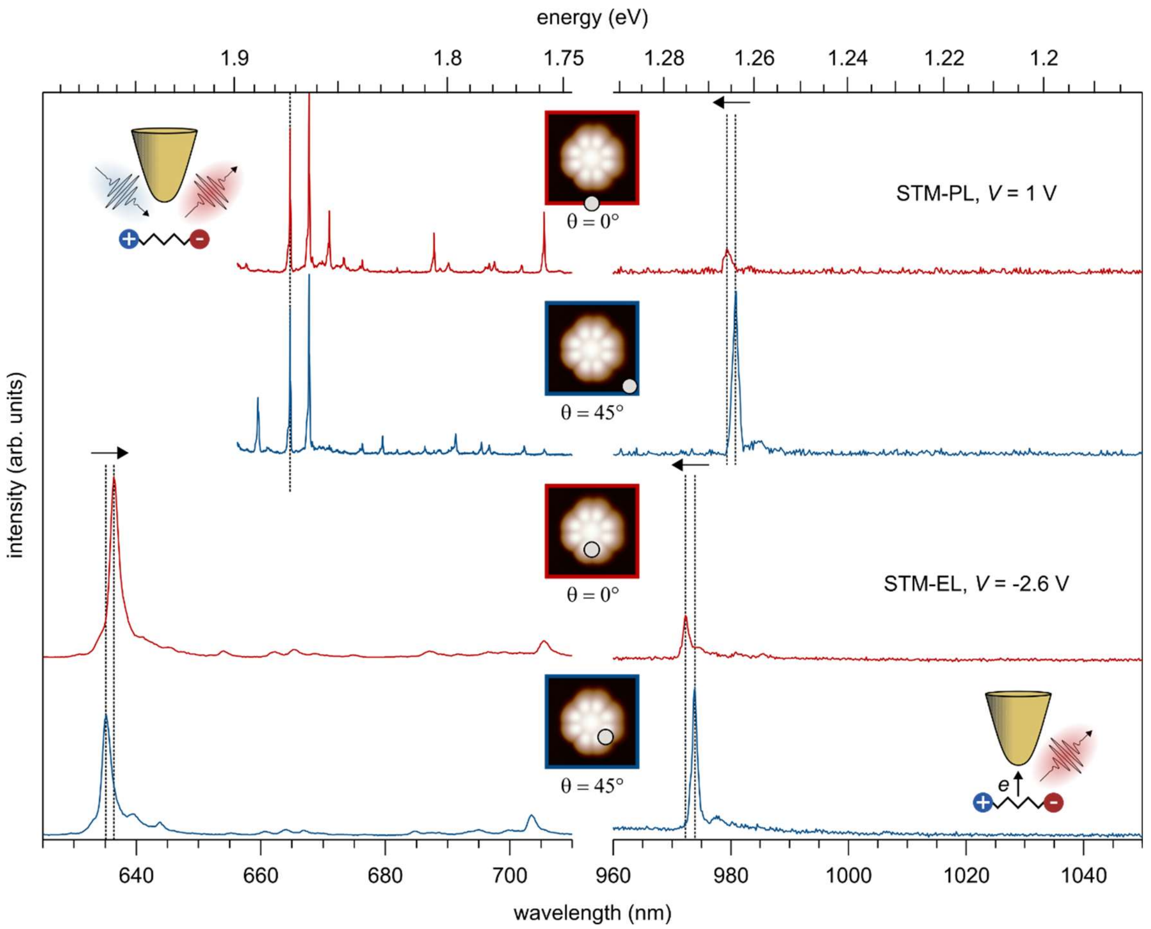

Supporting Figure 5. Angle dependence of fluorescence and phosphorescence. Bottom – STML spectra showing S_1 (left) and T_1 emission (right) obtained for azimuths θ = 0° and θ = 45°. Distance from the molecule r = 1.1 nm (I = 80 pA, V = −2.6 V; for S_1: t = 30 s, and for T_1: t = 120 s, grating: 300 grooves mm^{-1}). Top – TEPL spectra showing vibronic peaks (left) and T_1 emission (right) for r = 1.9 nm, i.e., off the molecular orbital (I = 3 pA, V = 1 V, t = 300 s, grating: 300 grooves mm^{-1}).

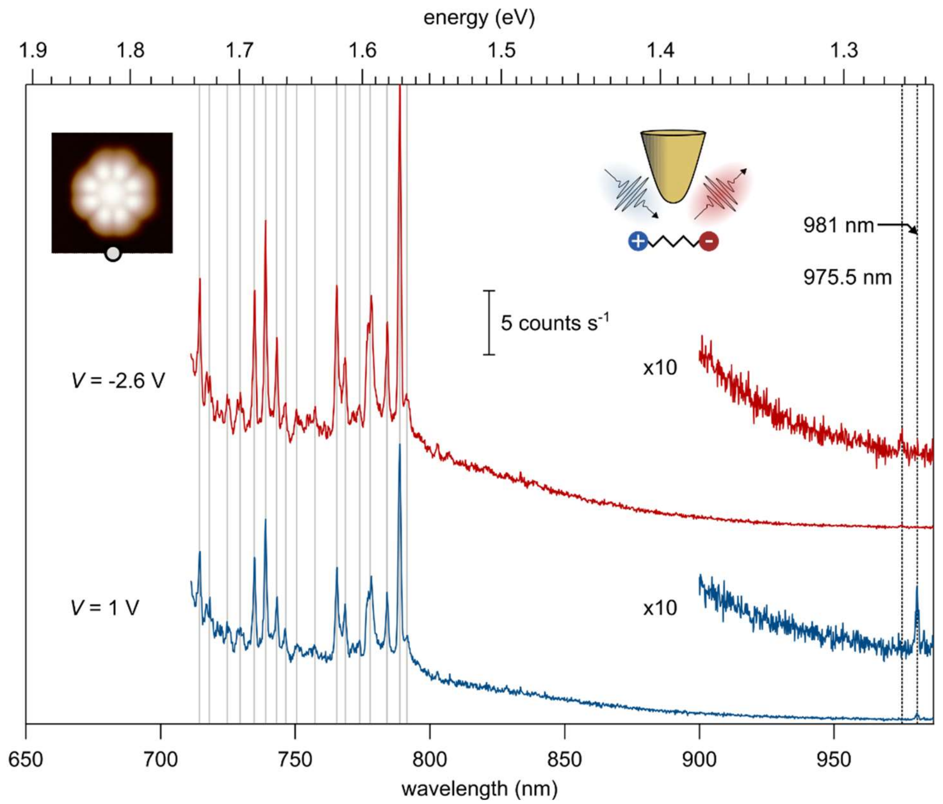

Supporting Figure 6. Extended TEPL spectra at different voltage polarities demonstrating the Stark shift of T_1 emission. Top – TEPL spectra at sample voltage $V = -2.6$ V (top) and +1 V (bottom) showing vibronic peaks and the T_1 emission line (laser power: 1µW, $I = 3$ pA, $t = 5$ min, grating: 300 grooves mm^{-1}). The blowups (×10) of the T_1 emission line demonstrate a voltage dependent redshift. Inset (top left): STM topography image of the molecule with a gray dot marking the tip-position (I = 3pA, V = −2.6V, size: 3.5×3.5 nm^2).

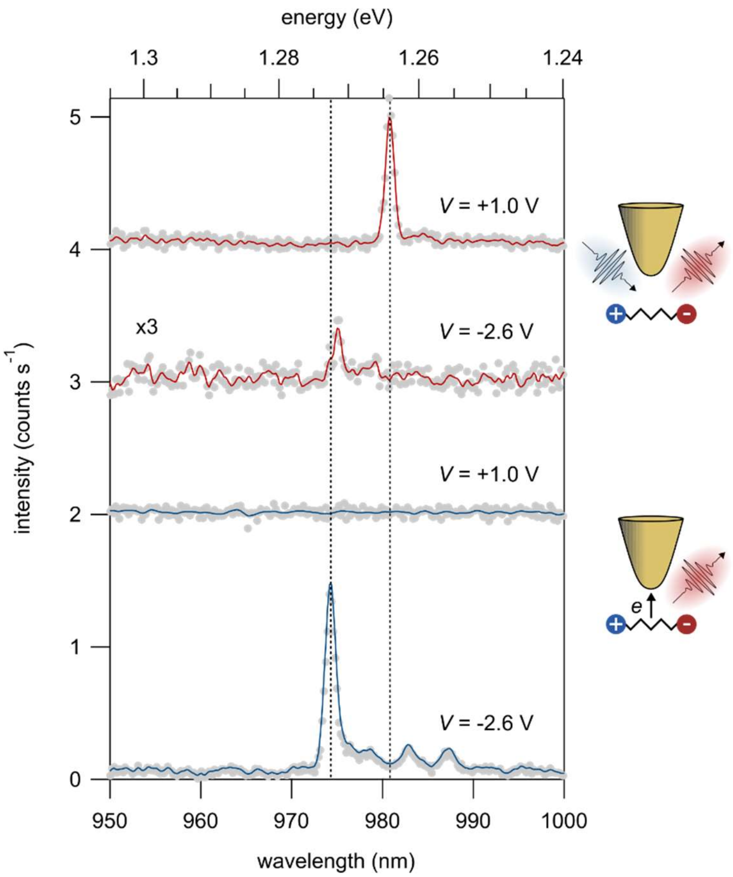

Supporting Figure 7. Voltage dependence of the T_1 emission line comparing STML and TEPL. Bottom – STML spectra at $V = -2.6$ V and +1 V ($I = 60$ pA, $t = 120$ s, $\theta = 0°$, $r = 1.1$ nm, grating: 300 grooves mm^{-1}). Top – TEPL spectra at $V = -2.6$ V and +1 V (laser power: 1 µW, $I = 3$ pA, $t = 300$ s, $\theta = 0°$, $r = 1.9$ nm, grating: 300 grooves mm^{-1}) showing the voltage dependent shift of the T_1 line.

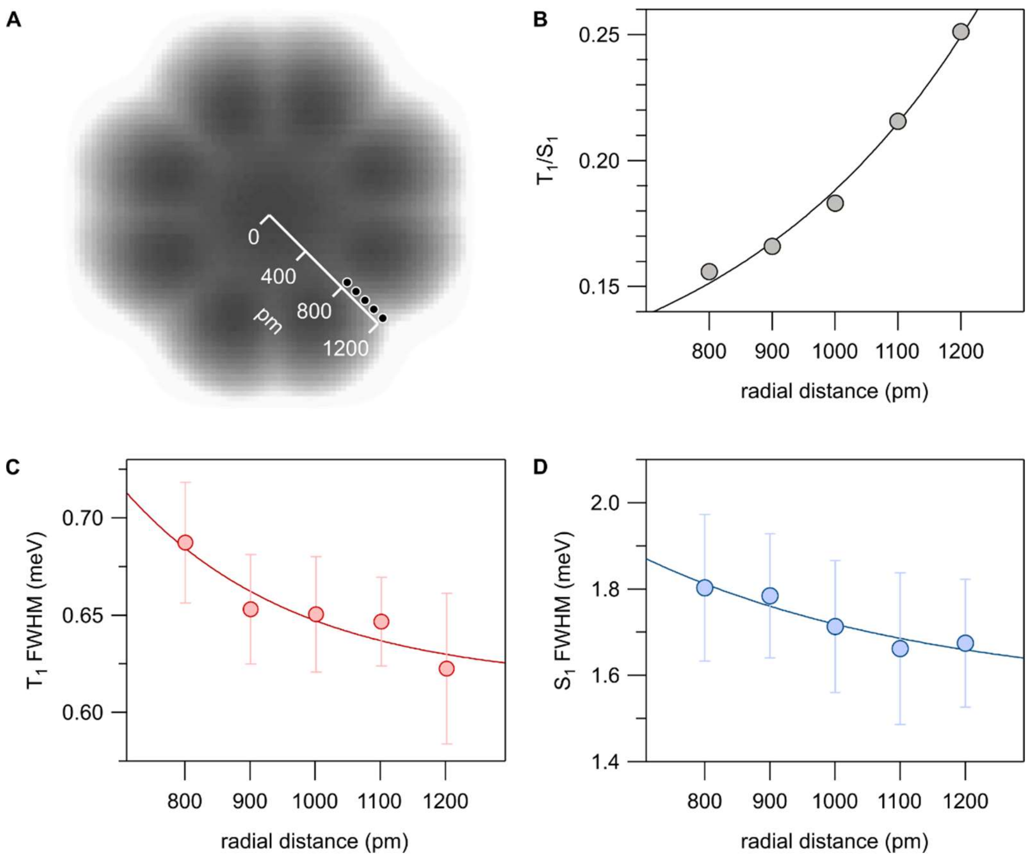

Supporting Figure 8. Radial distance-dependent X-P coupling (Purcell effect) of the S_1 and T_1 emissions. (A) STM topography of the molecule with tip positions marked for the data presented in B-D (I = 4 pA, V = -2.6 V). (B) T_1/S_1 intensity ratio as a function of radial distance from the molecule center (I = 50 pA, V = -2.6 V; for S_1: t = 10 s, and for T_1: t = 120 s; grating: 300 grooves mm^{-1}). (C, D) FWHM evaluated using a Lorentzian line shape fit to the T_1 and S_1 emission lines, respectively, for the same tip positions as in B. All solid lines are exponential fits to guide the eye.

References

(1) Yang, B.; Kazuma, E.; Yokota, Y.; Kim, Y. Fabrication of Sharp Gold Tips by Three-Electrode Electrochemical Etching with High Controllability and Reproducibility. *Journal of Physical Chemistry C* **2018**, *122* (29), 16950-16955. DOI: 10.1021/acs.jpcc.8b04078.

(2) Frisch, M. J.; Trucks, G. W.; Schlegel, H. B.; Scuseria, G. E.; Robb, M. A.; Cheeseman, J. R.; Scalmani, G.; Barone, V.; Petersson, G. A.; Nakatsuji, H.; et al. Gaussian 16 Rev. C.01. **2016**.

(3) Weigend, F.; Ahlrichs, R. Balanced basis sets of split valence, triple zeta valence and quadruple zeta valence quality for H to Rn: Design and assessment of accuracy. *Physical Chemistry Chemical Physics* **2005**, *7* (18), 3297-3305. DOI: 10.1039/b508541a.

(4) Rappoport, D.; Furche, F. Property-optimized Gaussian basis sets for molecular response calculations. *Journal of Chemical Physics* **2010**, *133* (13). DOI: 10.1063/1.3484283.

(5) Andrae, D.; Haussermann, U.; Dolg, M.; Stoll, H.; Preuss, H. Energy-adjusted *ab-initio* pseudopotentials for the 2nd and 3rd row transition-elements. *Theoretica Chimica Acta* **1990**, *77* (2), 123-141. DOI: 10.1007/bf01114537.

(6) Becke, A. D. Density-functional thermochemistry. III. The role of exact exchange. *Journal of Chemical Physics* **1993**, *98* (7), 5648-5652. DOI: 10.1063/1.464913.

(7) Baer, R.; Livshits, E.; Salzner, U. Tuned Range-Separated Hybrids in Density Functional Theory. In *Annual Review of Physical Chemistry*, Vol. 61; 2010; pp 85-109.

(8) Henderson, T. M.; Izmaylov, A. F.; Scalmani, G.; Scuseria, G. E. Can short-range hybrids describe long-range-dependent properties? *Journal of Chemical Physics* **2009**, *131* (4). DOI: 10.1063/1.3185673.

(9) Santoro, F.; Improta, R.; Lami, A.; Bloino, J.; Barone, V. Effective method to compute Franck-Condon integrals for optical spectra of large molecules in solution. *Journal of Chemical Physics* **2007**, *126* (8). DOI: 10.1063/1.2437197.

(10) Santoro, F.; Lami, A.; Improta, R.; Barone, V. Effective method to compute vibrationally resolved optical spectra of large molecules at finite temperature in the gas phase and in solution. *Journal of Chemical Physics* **2007**, *126* (18). DOI: 10.1063/1.2721539.

(11) Santoro, F.; Lami, A.; Improta, R.; Bloino, J.; Barone, V. Effective method for the computation of optical spectra of large molecules at finite temperature including the Duschinsky and Herzberg-Teller effect:: The Q_x band of porphyrin as a case study. *Journal of Chemical Physics* **2008**, *128* (22). DOI: 10.1063/1.2929846.

(12) Barone, V.; Bloino, J.; Biczysko, M.; Santoro, F. Fully Integrated Approach to Compute Vibrationally Resolved Optical Spectra: From Small Molecules to Macrosystems. *Journal of Chemical Theory and Computation* **2009**, *5* (3), 540-554. DOI: 10.1021/ct8004744.

(13) Scivetti, I.; Persson, M. Frontier molecular orbitals of a single molecule adsorbed on thin insulating films supported by a metal substrate: electron and hole attachment energies. *Journal of Physics-Condensed Matter* **2017**, *29* (35). DOI: 10.1088/1361-648X/aa7c3a.

(14) Neese, F. The ORCA program system. *Wiley Interdisciplinary Reviews-Computational Molecular Science* **2012**, *2* (1), 73-78. DOI: 10.1002/wcms.81.

(15) Neese, F. Software update: the ORCA program system, version 4.0. *Wiley Interdisciplinary Reviews-Computational Molecular Science* **2018**, *8* (1). DOI: 10.1002/wcms.1327.

(16) Neese, F.; Wennmohs, F.; Becker, U.; Riplinger, C. The ORCA quantum chemistry program package. *Journal of Chemical Physics* **2020**, *152* (22). DOI: 10.1063/5.0004608.

(17) Weigend, F. Accurate Coulomb-fitting basis sets for H to Rn. *Physical Chemistry Chemical Physics* **2006**, *8* (9), 1057-1065. DOI: 10.1039/b515623h.

(18) Hellweg, A.; Hättig, C.; Höfener, S.; Klopper, W. Optimized accurate auxiliary basis sets for RI-MP2 and RI-CC2 calculations for the atoms Rb to Rn. *Theoretical Chemistry Accounts* **2007**, *117* (4), 587-597. DOI: 10.1007/s00214-007-0250-5.

(19) Neese, F.; Wennmohs, F.; Hansen, A.; Becker, U. Efficient, approximate and parallel Hartree-Fock and hybrid DFT calculations. A 'chain-of-spheres' algorithm for the Hartree-Fock exchange. *Chemical Physics* **2009**, *356* (1-3), 98-109. DOI: 10.1016/j.chemphys.2008.10.036.

(20) Izsák, R.; Neese, F. An overlap fitted chain of spheres exchange method. *Journal of Chemical Physics* **2011**, *135* (14). DOI: 10.1063/1.3646921.

(21) van Wüllen, C. Molecular density functional calculations in the regular relativistic approximation:: Method, application to coinage metal diatomics, hydrides, fluorides and chlorides, and comparison with first-order relativistic calculations. *Journal of Chemical Physics* **1998**, *109* (2), 392-399. DOI: 10.1063/1.476576.

(22) Pantazis, D. A.; Chen, X. Y.; Landis, C. R.; Neese, F. All-electron scalar relativistic basis sets for third-row transition metal atoms. *Journal of Chemical Theory and Computation* **2008**, *4* (6), 908-919. DOI: 10.1021/ct800047t.

(23) Pantazis, D. A.; Neese, F. All-Electron Scalar Relativistic Basis Sets for the Lanthanides. *Journal of Chemical Theory and Computation* **2009**, *5* (9), 2229-2238. DOI: 10.1021/ct900090f.

(24) Pantazis, D. A.; Neese, F. All-Electron Scalar Relativistic Basis Sets for the Actinides. *Journal of Chemical Theory and Computation* **2011**, *7* (3), 677-684. DOI: 10.1021/ct100736b.

(25) Pantazis, D. A.; Neese, F. All-electron scalar relativistic basis sets for the 6*p* elements. *Theoretical Chemistry Accounts* **2012**, *131* (11). DOI: 10.1007/s00214-012-1292-x.

(26) Li, E. Y. T.; Jiang, T. Y.; Chi, Y.; Chou, P. T. Semi-quantitative assessment of the intersystem crossing rate: an extension of the El-Sayed rule to the emissive transition metal complexes. *Physical Chemistry Chemical Physics* **2014**, *16* (47), 26184-26192. DOI: 10.1039/c4cp03540b.

(27) de Souza, B.; Farias, G.; Neese, F.; Izsák, R. Predicting Phosphorescence Rates of Light Organic Molecules Using Time-Dependent Density Functional Theory and the Path Integral Approach to Dynamics. *Journal of Chemical Theory and Computation* **2019**, *15* (3), 1896-1904. DOI: 10.1021/acs.jctc.8b00841.

(28) Neese, F. Efficient and accurate approximations to the molecular spin-orbit coupling operator and their use in molecular *g*-tensor calculations. *Journal of Chemical Physics* **2005**, *122* (3). DOI: 10.1063/1.1829047.

(29) Hirata, S.; Head-Gordon, M. Time-dependent density functional theory within the Tamm-Dancoff approximation. *Chemical Physics Letters* **1999**, *314* (3-4), 291-299. DOI: 10.1016/s0009-2614(99)01149-5.

(30) Schatz, G. C.; Ratner, M. A. *Quantum Mechanics in Chemistry*; Dover Publications Inc. , 2002.

(31) Kaiser, K.; Rosławska, A.; Romeo, M.; Scheurer, F.; Neuman, T.; Schull, G. Electrically driven cascaded photon-emission in a single molecule. *Physical Review X* **2025**, *15* (2). DOI: doi.org/10.1103/PhysRevX.15.021072.

(32) Menzel, E. R.; Rieckhoff, K. E.; Voigt, E. M. Dynamics of triplet-state of phthalocyanine complexes of platinum metals in zero-field. *Journal of Chemical Physics* **1973**, *58* (12), 5726-5734. DOI: 10.1063/1.1679197.
